# An ethnobiological study on traditional knowledge associated  with black-boned sheep (*Ovis aries*) in Northwest Yunnan, China

**DOI:** 10.1186/s13002-022-00537-5

**Published:** 2022-05-17

**Authors:** Yanxiao Fan, Zhuo Cheng, Bo Liu, Xian Hu, Maroof Ali, Chunlin Long

**Affiliations:** 1 Key Laboratory of Ecology and Environment in Minority Areas (Minzu University of China), National Ethnic Affairs Commission, Beijing, 100081 China; 2 College of Life and Environmental Sciences, Minzu University of China, Beijing, 100081 China; 3 College of Life Science, Anhui Normal University, Wuhu, 241000 China; 4 Key Laboratory of Ethnomedicine (Minzu University of China), Ministry of Education, Beijing, 100081 China; 5 Kunming Institute of Botany, Chinese Academy of Sciences, Kunming, 650201 China

**Keywords:** Black-boned sheep, Genetic resources, Forage plants, Pumi people, Traditional knowledge

## Abstract

**Background:**

Black-boned sheep is a precious genetic resource with black quality traits cultivated by the Pumi people in Tongdian Town, Lanping County, Nujiang Lisu Autonomous Prefecture, Northwest Yunnan, China. It has been included in the “National Breed List of Livestock and Poultry Genetic Resources.” The local communities have a deep understanding of black-boned sheep. The traditional knowledge of black-boned sheep is essential to their conservation and sustainable development. In spite of this, there was no information on traditional knowledge associated with black-boned sheep so far. The aim of this study wasaimed to document traditional knowledge and culture, to elucidate information about forage plants, and to investigate the conservation strategy of black-boned sheep.

**Method:**

Four field surveys were conducted from July 2019 to May 2021. A total of seven villages and the Pumi Culture Museum in Lanping County are being investigated. A semi-structured interview method was used to interview 67 key informants. During the investigation, we also participated in the grazing activities of black-boned sheep, observed the appearance characteristics and the herd structure of the black-boned sheep, and demonstrated traditional knowledge regarding black-boned sheep, including grazing methods, forage plants, and related customs and habits.

**Results:**

We assumed that a majority of people in the current study sites were able to could distinguish black-boned sheep from their relatives by their black bones, blue-green gums, and blue-purple anus. The local people manage their black-boned sheep based on the number of sheep by sex, age, and role in a flock in the different breeding environments. Different grazing strategies have been adopted in different seasons. Through ethnobotanical investigations, 91 species of forage plants in 30 families were identified, including herbaceous, shrubs, lianas, and trees. Among all the plant species consumed by the black-boned sheep, Rosaceae species make up the greatest number, with 16, followed by Asteraceae, with 9, and 8 species of Fabaceae and Poaceae. Considering the abundance of forage plants and the preference for black-boned sheep, *Prinsepia utilis* and the plants of *Rubus*, *Berberis*, and *Yushania* occupy dominant positions. Plants used for foraging are divided into two categories: wild and cultivated. Due to the lack of forage plants in fall and winter, the local people mainly cultivate crops to feed their black-boned sheep. In addition, the black-boned sheep is an influential cultural species in the local community and plays a prominent role in the cultural identity of the Pumi people.

**Conclusion:**

Sheep play an essential role in the inheritance of the spiritual culture and material culture of the Pumi ethnic group. The formation of the black-boned sheep is inseparable from the worship of sheep by the Pumi people. With a long-term grazing process, the locals have developed a variety of traditional knowledge related to black-boned sheep. This is the experience that locals have accumulated when managing forests and grasslands. Therefore, both the government and individuals should learn from the local people when it comes to protecting black-boned sheep. No one knows black-boned sheep better than them. The foremost evidence of this is the rich traditional knowledge of breeding black-boned sheep presented by key informants.

## Introduction

Since prehistoric times, domesticated livestock, and poultry have played an essential role in human societies. The survival and development of small-scale farmers in many parts of the world are closely related to domestic animals [1–5]. On the one hand, domestic animals, especially livestock ruminants, provide labor and fertilizer for small-scale farmers, helping them integrate and efficiently use limited land resources [6–8]. In addition to providing nutrition, domestic animals also generate income for small-scale farmers in the forms of meat, eggs, milk, etc. [9, 10]. Over the past decade, animal husbandry has shown much closer relationships with several other fields. These include food security, biodiversity conservation, environmental protection, and rural poverty alleviation [11–17]. Earlier studies demonstrated that livestock and poultry genetic resources in minority areas could serve as a major strategic resource for a nation and contribute significantly to global biodiversity [18–21]. As a consequence, many countries are placing a greater emphasis on developing animal husbandry. In one aspect, the government will protect rare domestic animal resources by issuing relevant policies and financial incentives. Contractually, vaccination campaigns for domestic animals and the professional knowledge training for breeders must be strictly adhered to [22–26]. However, research shows that the development of animal husbandry fundamentally relies on the original breeding environment of domestic animals. The most appropriate breeding methods could only be determined by combining the traditional knowledge of indigenous people with scientific knowledge. In sum, we can summarize the forage plant species that indigenous people use in raising a certain type of livestock. We can also build a database of relevant forage plants, and assess the selection preference of this domestic animal for forage. Then, the most suitable feed can be selected when developing the related breeding industry [2, 7, 8]. A number of studies related to this have been conducted around the world [6–9, 27–29].

China has a huge land area, diverse topography and landforms, an abundance of natural resources, and a long history of the domestication of livestock and poultry. There is a rich cattle culture in China, which overlaps deeply related with traditional customs, farming life, and cognitive psychology, where cattle cover *Bos* and *Bulalus*. This has resulted in a rich diversity of livestock and poultry resources throughout the country [30, 31]. China is one of the countries with the most extensive livestock and poultry genetic resources in the world. Due to its wide geographical distribution and uneven protection of livestock and poultry resources, China is also one of the countries where livestock and poultry genetic resources are seriously threatened [32, 33]. In general, China’s local livestock and poultry resources are generally showing a downward trend. Many local breeds are on the verge of extinction, and some have even become extinct, making the situation of local livestock and poultry breed resources not optimistic [34, 35]. Hence, they need to be protected with proper strategies.

Black-boned sheep is a unique genetic resource of livestock that has been raised and domesticated by the Pumi people in Tongdian, a township in Lanping County, Yunnan Province, for a long time. It is considered to be the second animal with heritable characteristics of melanin in the world besides the black-boned chicken, and it is the only mammal that has been found to contain a large amount of melanin in the body so far [36–39]. The most direct manifestations of the black-bone trait of the black-boned sheep are the black periosteum and black meat. Several studies have demonstrated that the black-boned sheep’s black trait is caused by a high content of melanin, which is heritable and a very rare genetic resource. Moreover, the high melanin content of black-boned sheep is of a substantial benefit to medicine and health [40–42]. Since the black-boned sheep was first discovered in 2001, it has attracted much attention. In 2006, the country officially named the sheep as “Lanping black-boned sheep” according to its origin and characteristics, which was identified and approved by the expert group of the National Animal and Poultry Genetic Resources Appraisal Committee in October 2009. In the same year, it was listed in the “National List of Animal and Poultry Genetic Resources,” “China Rare Animal Breeds List” and “World Rare Animal Species List” [43, 44]. Even though black-boned sheep have always been of high concern to the state, protecting them has always been a challenging issue. The practice has proved that the protection of genetic resources cannot be separated from the natural and cultural environment in which they are located, as is the case with black-boned sheep [45–47].

The Pumi people in Lanping have developed a lot of traditional knowledge relating to black-boned sheep, including how to distinguish them, how to graze, and what they like to eat. In addition, sheep are an intrinsic component of the indigenous culture of the Pumi people. Although the above local knowledge is vital for the protection of black-boned sheep, there was no mention of traditional knowledge about black-boned sheep. Therefore, in this study, we aimed to (1) investigate the traditional knowledge of the Pumi people on raising black-boned sheep; (2) to document the wild plants that the shepherd prefers to use for feeding black-boned sheep, and the crops used by the shepherd as supplementary forage in the winter; (3) to assess the field abundance of forage plants as well as the preference of black-boned sheep for forage plants; and (4) to record the local customs, stories, and legends related to sheep.

## Methods

### Study area

Tongdian Township is located at the southernmost end of the World Natural Heritage Protection Area “Three Parallel Rivers,” with a complete ecosystem and rich biodiversity. The Tongdian covers a total area of 521.33 km^2^, the highest elevation is 3688 m, the lowest elevation is 2237 m, the annual average temperature is 10.7 °C, the annual average accumulated temperature is 2840 °C, and the annual average rainfall is about 1024.1 mm. Tongdian is home to 9 ethnic groups, including Bai, Pumi, Yi, and Lisu. Due to limited communication with the outside world, local ethnic groups still retain the habit of planting old varieties of crops and raising old varieties of poultry and livestock [48–51]. Among them, the black-boned sheep domesticated and raised by the Pumi is a rare genetic resource in the world [38, 39].

Pumi is one of the ethnic groups with a long history and ancient culture in China. They mainly live in Lanping County in Nujiang Prefecture, Ninglang and Yulong County in Lijiang City, and Weixi County in Diqing Prefecture [52–54]. Pumi people were originally nomads, proficient at raising and grazing animals, and animal husbandry occupies an active position in their production and life. Moreover, gathering and hunting are also part of the Pumi’s socio-economic life. Therefore, the Pumi has a broad understanding of the diversity and nutritional potential of local plants. However, because the Pumi people only have language and no written words, they can only transmit and exchange information verbally [55–57]. This lack of written records means that local traditional knowledge is particularly prone to loss and extinction. Consequently, there is an urgent need to record indigenous knowledge related to this ethnic group.

Black-boned sheep have been raised by Pumi people for a long time and they rarely exchange sheep with the outside world. They commonly grow at an altitude of about 1900–2800 m, and the breeding range is within 30 km of Tongdian. With economic development, sheep breeds from outside have been introduced, which not ﻿only threatens the integrity of the native black-boned sheep but also reduces the number of farmers who breed black-boned sheep. To protect black-boned sheep, the local government encourages local companies to set up breeding plants to breed black-boned sheep. However, what kind of feed is suitable for black-boned sheep and what breeding methods can ensure the high quality of black-boned sheep require further investigation.

### Ethnobiological survey

**Fig. 1:**
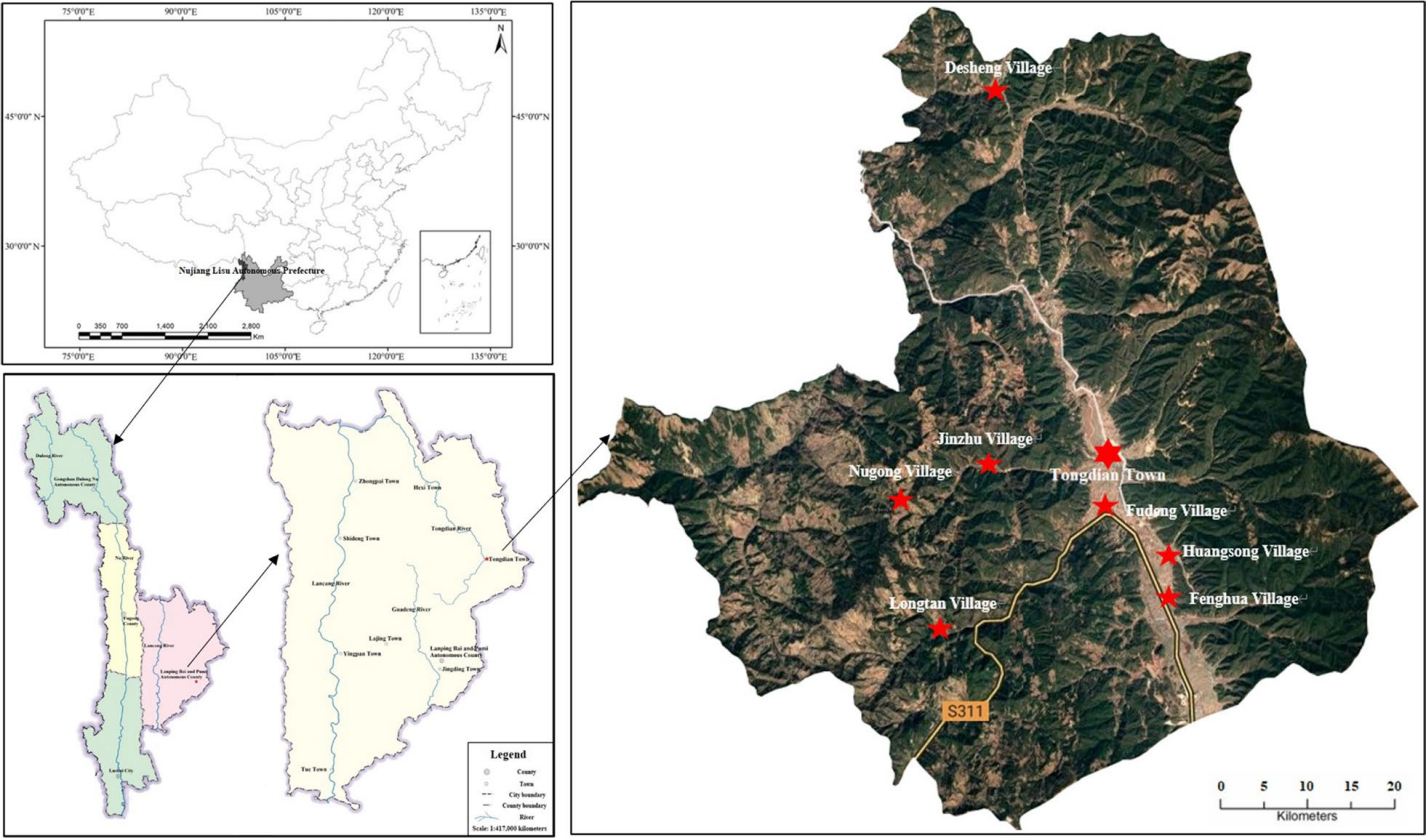
The study area in Tongdian, Lanping of Yunnan, China

Our ethnobotanical survey was mainly carried out in Tongdian Town, the original habitat of black-boned sheep. We conducted three surveys in Desheng, Longtan, Fenghua, Jinzhu, Huangsong, Fudeng, Nugong villages in Tongdian Town, and Pumi Culture Museum in Lanping County from 2019 to 2021 (Fig. 1). Before the investigations, all reporters were informed of the purpose of the investigations and obtained their consent. Methods to collect data include free listing, semi-structured interviews, and participatory observation. A total of 67 key informants were screened using the snowball sampling method, including 48 males and 19 females, with an average age of 57 years old [58, 59].

**Fig. 2:**
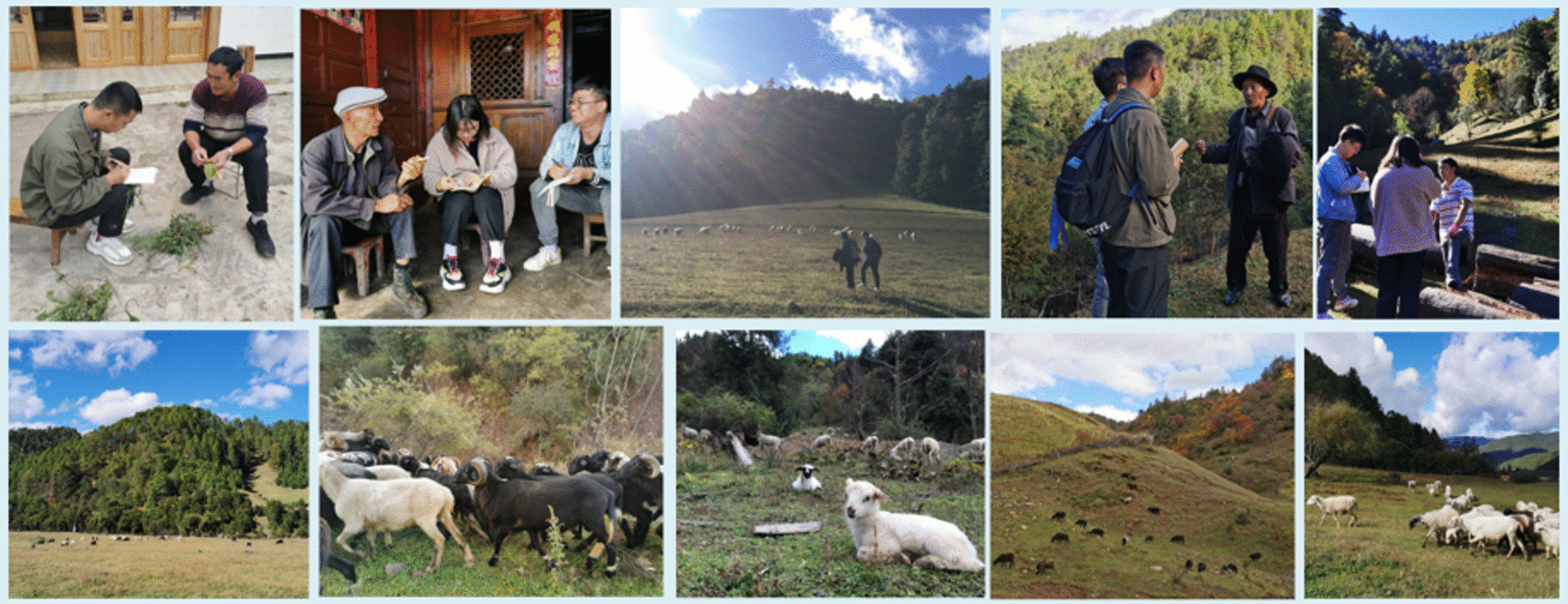
Field surveys and grazing areas of black-boned sheep (Photographed by Chunlin Long in November 2020)

Semi-structured interviews are conducted in response to the questions in Table 1. A participatory observation was conducted with the locals during the grazing activities. During this process, forage plant specimens are collected and the forage feeding of the black-boned sheep in their wild habitat is observed (Fig. 2). Nomenclature of all vascular plants was followed *Flora of China*, and World Flora Online (www.worldfloraonline.org) as well. Professor Chunlin Long and Dr. Bo Liu identified the plant species. The voucher specimens were deposited in the Herbarium of the College of Life and Environmental Sciences, Minzu University of China, in Beijing [60].

**Table 1:** Questions used for semi-structured interviews

No	Question
1	Why do you like raising black-boned sheep?
2	Is the breeding of black-boned sheep mainly grazing or captive breeding?
3	Which plants do black-boned sheep eat?
4	What are the local names of the plants consumed by black-boned sheep?
5	What is the favorite plant for black-boned sheep?
6	Will there be changes in the eating habits of black-boned sheep in different growth stages?
7	Where do black-boned sheep usually like to move?
8	When there is a shortage of forage, what do black-boned sheep mostly eat?
9	What are the main threats facing the black-boned sheep?
10	Is there any traditional culture related to black-boned sheep in the local area?

### Data analysis

All data information is based on the first-hand information provided by 67 key informants, including the used parts of forage plants, the number of forage reserves in the wild, the preference of black-boned sheep, etc. (also see items 3 and 5 in Table 1). Regarding the abundance of forage plants in the field, we evaluated the frequency of encountering the plants during the investigation. In this area, if we encountered a plant species only 1–2 times, this indicates a low abundance 3–4 times for medium abundance, and more than 4 times (5 and over) for high abundance. The preference for forage was evaluated by the number of times recommended by key informants. The more frequently this is mentioned, the more black-boned sheep will flock here to graze.

## Results and discussion

### How to distinguish black-boned sheep and form a flock

From a morphological point of view, the black-boned sheep is similar to Tibetan sheep in that it has a wide chest, straight back, large abdomen but not drooping, relatively short body, strong limbs, short tail, and conical shape. The head and limbs are poorly covered with wool and the coat is thick. The blackness of the black-boned sheep has nothing to do with the color of the coat. The older an individual is, the deeper the blackness will be. The difference between black-boned sheep and other sheep is that the eye conjunctiva is brown, the skin of the elbow joints of the forelegs and hind limbs is purple, the skin of the hair roots and underarms of the white wool black-boned sheep is purple, the mouth and tongue are bright black, the gums are blue-green, and the anus is blue-purple. These characteristics of black-boned sheep allow local people to distinguish them from other sheep.

According to key informants, if the flock of black-boned sheep can be organized reasonably during grazing, it will save labor and facilitate the management of the flock. The local communities must determine the number, sex, and age composition of the black-boned sheep based on the breeding area and grazing environment. If the place has a large pasture or pasturing area, the proper number of black-boned sheep is 400–450, of which 200–250 ewes are used to breed lambs and breed as mutton sheep, 150–200 rams are castrated, as a meat sheep fattening, and the remaining 50–100 rams are used to breed ewes. However, if there is no large area of pasture in the area, the number of sheep should not be too big, usually around 100.

Black-boned sheep are accustomed to moving on hillsides and dense forests, looking for food everywhere (Fig. 2). A large amount of activity makes the black-boned sheep stronger and stronger. Therefore, most of the time, the black-boned sheep do not need to be looked after. In the winter, when the mountains are covered in snow and rain, they need warm houses. In Pumi communities, sheepfolds are generally rebuilt from dilapidated old houses, or built with materials available everywhere, such as stones, branches, and bamboo, at low cost. Shepherds are usually 1 m high to prevent them from escaping beyond the pen. According to demand, dry crop straw and hay can be laid in the pen in winter to help the black-boned sheep keep warm.

### Selection of grazing sites and use of black-boned sheep

The Pumi people do not have their own specific pastures. The mountain forests, grasslands, and wasteland around the villages can become natural pastures for black-boned sheep, and farmers in the same village share these resources. The Pumi people distinguish their sheep when grazing by putting bells on their necks so they do not get confused with others' sheep. They also mark head sheep’s bodies. As long as the head sheep is mastered, the other sheep will not run around. According to key informants, the flock also recognizes the voice of its owner. When grazing, the local people will consciously change their positions constantly to prevent the sheep from over-grazing on the vegetation in an area and causing grassland degradation. Moreover, the high mobility of the black-boned sheep further causes it to constantly change its feeding location. The local government will also fence off the pastures in a certain area and close them for a few seasons for the purpose of restoring vegetation.

For the Pumi people, the black-boned sheep is a treasure. The wool is used for spinning, weaving, and making their traditional clothing. The sheepskin can be incorporated into a felt hat for the Pumi people to keep warm in winter. Its milk can also be made into a variety of dairy snacks. But the most important thing for the Pumi people is the meat of the black-boned sheep, which is not only an important source of income for them, but serves as a significant source of protein for maintaining physical strength. In the Pumi community, it is very common to consume mutton. In addition to eating fresh, black-boned mutton can also be preserved by processing. Most of the black-bone sheep they raise are sold to buyers from various places, and then appear on the markets in the form of mutton. Importantly, sheep manure produced by black-boned sheep in captivity in winter is a highly effective natural fertilizer. As part of their gardening practices, the Pumi people usually spread it evenly on the vegetable fields in front of and behind their houses.

### Indigenous knowledge about black-boned sheep grazing

Various problems need to be addressed during different grazing seasons. These issues are also the criteria for testing whether a shepherd is qualified.

**Fig. 3:**
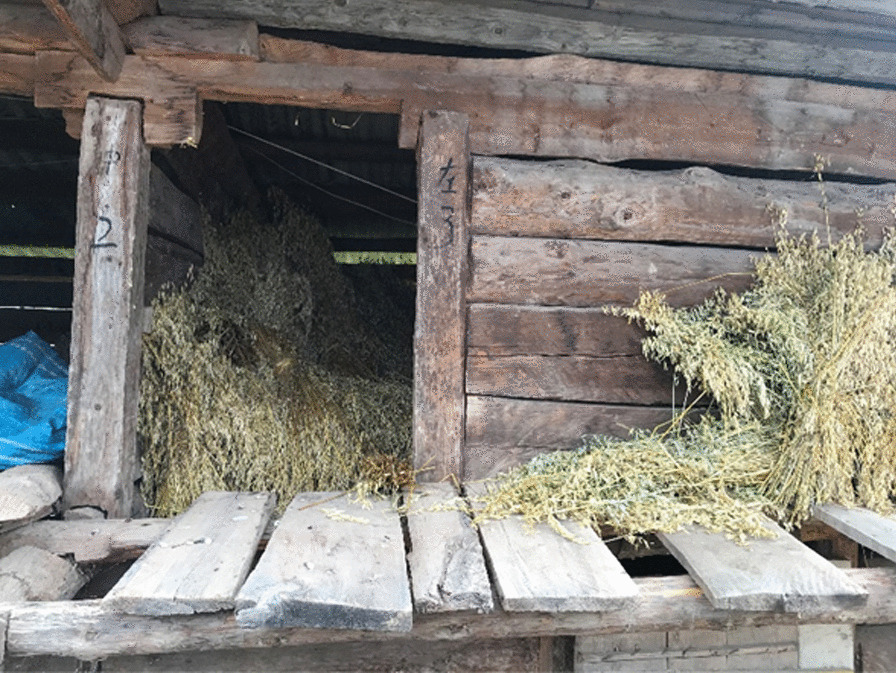
A hay house with dry ground parts of *Avena sativa* L. stacked (Photographed by Yanxiao Fan in November 2020)

It is not advisable to graze too early in spring. On the one hand, it is cold in the morning, and the new shoots of the forage grass have high moisture content and carry dew. If the sheep eat too much, it will cause diarrhea. On the other hand, sheep graze on hay throughout the winter. They will become greedy when they see fresh green grass. Under the leadership of the leader, the sheep will run around looking for grass to eat. This will not only cause the sheep to feed on grass without growing meat but also cause poisoning due to accidental eating of poisonous weeds. In addition, eating too much grass will also lead to indigestion and flatulence. In this regard, there is a famous proverb in the local community: “stop the leader, the sheep will be fat and strong; let the leader go, the sheep will not grow fat.” To avoid these situations, the locals will feed some hay to the sheep before they start grazing, and then let the sheep move freely. The hay is usually the dried aerial parts of crops like *Avena sativa* L., *Pisum sativu*m L., etc., which are harvested and threshed in the summer or autumn, and stacked in wooden houses (Fig. 3).

Summer is hot and rainy, so grazing activities should follow the guidelines of starting out early and returning late to prevent heatstroke in the flock. At noon, let the flock rest in a ventilated and shaded place to prevent the flock from getting together, and provide the sheep with more water.

Autumn is the season for sheep to gain weight so they can survive the cold winter. At this time, the pasture is abundant, and there are also many mature wild fruits to help the sheep improve their diet and supplement nutrition. Moreover, autumn is also the peak season for sheep to ovulate and mate. Therefore, the shepherd should focus on letting the sheep eat enough and well, breeding for sale, safe overwintering, and offspring reproduction.

As the winter turns cold, the plants begin to wither and are accompanied by rain, snow, and frost. When grazing, it is important to keep lambs warm, prevent them from getting cold, and to keep them healthy. The sheep usually graze near a village or farmland, where leaves and hay are available for them to eat. When the weather is fine, the sheep can be properly basked in the sun, but do not allow pregnant ewes to exercise vigorously. In addition, the sheep shed must be properly maintained before the snow arrives.

In addition, it is essential to feed the sheep regularly with crushed hay feed mixed with salt and lard. According to locals, this can help digestion, increase appetite, and supplement nutrition. When grazing, it is also necessary to ensure that the sheep are allowed to drink water at least once a day, preferably from a mountain spring.

### The diversity of plants consumed by black-boned sheep

**Fig. 4:**
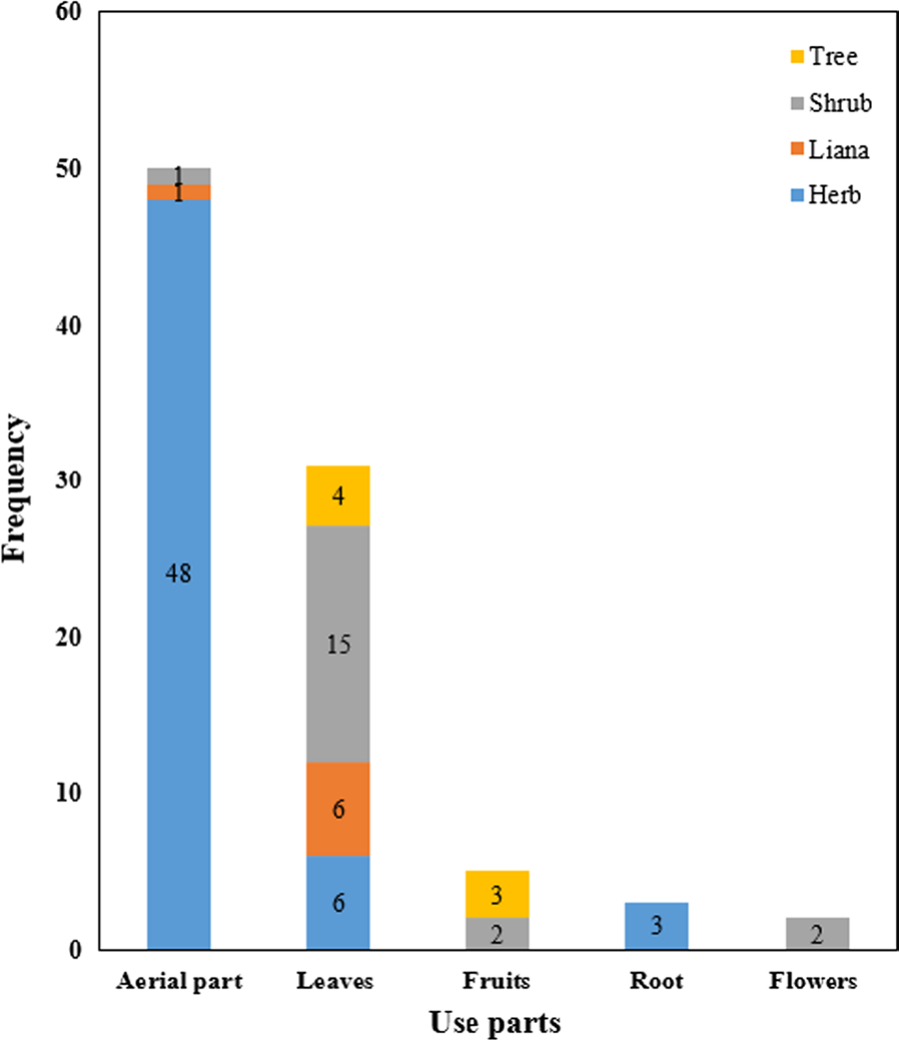
Frequently cited families of forage plant species

**Fig. 5:**
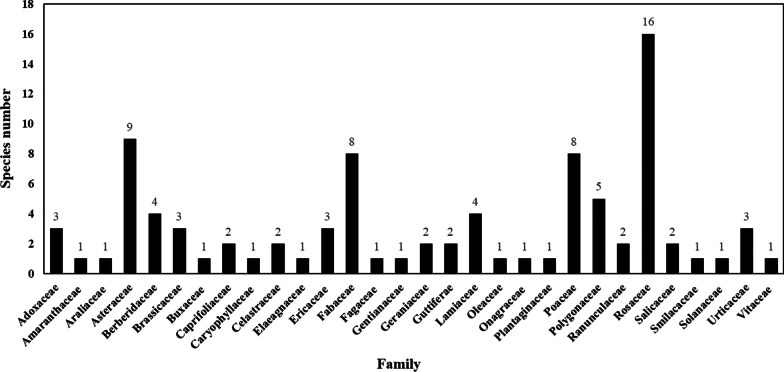
Plant parts consumed and the life forms of forage plants

The shepherd has rich knowledge of plant species or plant parts preferred to eat by black-boned sheep. In our research, we documented that black-boned sheep consumed forage plants. A total of 91 forage plants were recorded (Table 2), including 57 species of herbaceous plants, 20 species of shrubs, 7 species of lianas, and 7 species of trees (Fig. 4). These 91 species of forage plants belong to 30 families (Fig. 5). Most of them belong to the Rosaceae, with 16 species, including herbaceous, shrubs, and trees. Such as species of *Potentilla*, *Rubus*, and *Rosa*. The next group is the Asteraceae, with 9 species, and all of them are herbs. There are 8 species of Fabaceae and Poaceae. The parts of forage plants consumed by black-boned sheep include aerial parts, leaves, fruits, roots, and flowers (Fig. 4). Of which the aerial part accounts for 55%. Next is the leaves, which make up for 34%. Followed by fruit, roots, and flowers, accounting for 5.5%, 3.3%, and 2.2%, respectively. Those forage plants can be divided into two types: wild and cultivated. Cultivated plants are mainly used as supplementary feed in winter when wild forage plants are scarce. On the other hand, considering the abundance of forage plants and the preference of black-boned sheep, *Prinsepia utilis*, *Rosa multiflora*, and the plants of *Rubus*, *Berberis*, and *Yushania* occupy dominated positions. Many forage plants have various uses and are used by local people as food, medicine, decoration, nectar source, and green fertilizer.

**Table 2:** Fodder plant species consumed by black-boned sheep in Tongdian, Lanping of Yunnan, China

Voucher No	Family	Scientific name	Local name	Parts consumed	Life form	Consumed time	Preference	Abundance	Other purposes
TD007	Adoxaceae	*Sambucus adnata* Wall. ex DC.	Chu gao	Aerial part	Herb	All available time	**	**	Medicine for people
FD049	Adoxaceae	*Sambucus javanica* Blume	Si ai sa	Aerial part	Herb	All available time	**	**	Medicine for people
FD067	Adoxaceae	*Viburnum betulifolium* Batal.	Shi ji si	Fruits	Shrub	All available time	***	**	Fruits for people
TD013	Amaranthaceae	*Achyranthes bidentata* Blume	Chan gua zi	Aerial part	Herb	All available time	*	**	Medicine for people
FD086	Araliaceae	*Aralia chinensis* L.	Chu wu ji	Leaves	Shrub	All available time	**	**	Food for people
TD008	Asteraceae	*Anaphalis yunnanensis *(Franch.) Diels	Fu peng	Aerial part	Herb	All available time	*	***	Medicine for people
FD018	Asteraceae	*Arctium lappa* L.	Yang ba zi	Leaves	Herb	All available time	*	**	Medicine for people
FD087	Asteraceae	*Artemisia lavandulifolia *Candolle	Hei ke	Aerial part	Herb	All available time	**	***	Medicine for people
TD018	Asteraceae	*Bidens pilosa* L.	/	Aerial part	Herb	All available time	*	***	Medicine for people
FD054	Asteraceae	*Cirsium japonicum* Fisch. ex DC.	Eng qi ma qi	Leaves	Herb	All available time	*	***	Medicine for people
FD063	Asteraceae	*Galinsoga parviflora* Cav.	Yang kong zi	Aerial part	Herb	All available time	**	***	Medicine for people
TD014	Asteraceae	*Pseudognaphalium affine* (D. Don) Anderberg	/	Aerial part	Herb	All available time	*	***	Medicine for people
TD017	Asteraceae	*Senecio scandens* Buch-Ham. ex D. Don	Mia ni hen	Aerial part	Herb	All available time	*	***	Medicine for people
FD064	Asteraceae	*Taraxacum mongolicum* Hand-Mazz.	/	Leaves	Herb	All available time	**	***	Food, medicine for people
TD012	Berberidaceae	*Berberis diaphana* Maxim.	Zu da da	Leaves	Shrub	All available time	***	***	Medicine for people
FD074	Berberidaceae	*Berberis pruinosa* Franch.	Huang lian shu	Leaves	Shrub	All available time	***	***	Medicine for people
TD009	Berberidaceae	*Berberis* sp.	Xie zhu	Leaves	Shrub	All available time	***	***	Medicine for people
TD011	Berberidaceae	*Berberis tomentulosa* Ahrendt	Wa lu chi	Leaves	Shrub	All available time	***	***	Medicine for people
TD020	Brassicaceae	*Brassica juncea* var. *napiformis *Pailleux et Bois	Wei li bu	Roots	Herb	Winter only	**	***	Crop for people
TD021	Brassicaceae	*Brassica rapa* L.	/	Roots	Herb	Winter only	**	***	Crop for people
TD019	Brassicaceae	*Capsella bursa-pastoris* (L.) Medic.	/	Aerial part	Herb	Winter only	**	***	Crop for people
TD015	Buxaceae	*Sarcococca ruscifolia* Stapf	Xie xie seng	Leaves	Shrub	All available time	*	***	Medicine for people
FD014	Caprifoliaceae	*Dipsacus asper* Wallich ex Candolle	A ji ba mao	Aerial part	Herb	All available time	*	***	Medicine for people
FD021	Caprifoliaceae	*Lonicera yunnanensis* Franch.	Re na da	Leaves	Liana	All available time	**	**	Medicine, ornament for people
TD016	Caryophyllaceae	*Myosoton aquaticum* (L.) Moench	Ba ta na	Aerial part	Herb	All available time	*	***	Medicine for people
TD041	Celastraceae	*Celastrus angulatus* Maxim.	Ba da	Leaves	Liana	All available time	***	*	Ornament for people
TD010	Celastraceae	*Celastrus orbiculatus* Thunb.	Gua ning su	Leaves	Liana	All available time	***	*	Medicine for people
FD004	Elaeagnaceae	*Elaeagnus umbellata* Thunb.	Man xu zi	Fruits	Tree	All available time	***	**	Fruits for people
FD055	Ericaceae	*Rhododendron* decorum subsp. decorum	Sang si ding bao gua mi	Flowers	Shrub	All available time	**	***	Food for people
FD056	Ericaceae	*Rhododendron racemosum* Franch.	Dan bai zi	Flowers	Shrub	All available time	**	***	Ornament for people
FD043	Ericaceae	*Vaccinium fragile* Franch.	Zho he zi	Fruits	Shrub	All available time	**	**	Fruits for people
TD036	Fabaceae	*Astragalus sinicus* L.	/	Aerial part	Herb	All available time	***	***	Nectar source, green manure for people
TD029	Fabaceae	*Hylodesmum podocarpum *(Candolle) H. Ohashi & R. R. Mill	/	Aerial part	Herb	All available time	***	***	Nectar source, green manure for people
TD025	Fabaceae	*Medicago sativa* L.	/	Aerial part	Herb	All available time	***	***	Nectar source, green manure for people
TD049	Fabaceae	*Phaseolus vulgaris* L.	/	Aerial part	Herb	Winter only	***	***	Crop for people
TD051	Fabaceae	*Pisum sativum* L.	/	Aerial part	Liana	Winter only	***	***	Crop for people
TD026	Fabaceae	*Trifolium pratense* L.	/	Aerial part	Herb	All available time	***	***	Green manure for people
TD046	Fabaceae	*Trifolium repens* L.	/	Aerial part	Herb	All available time	***	***	Green manure for people
FD038	Fabaceae	*Vicia cracca* Benth.	Lu fei	Aerial part	Herb	All available time	***	***	Nectar source, green manure for people
FD052	Fagaceae	*Quercus aquifolioides* Rehd. et Wils.	Fu qi zi	Leaves	Tree	All available time	**	**	/
FD044	Gentianaceae	*Gentiana macrophylla* Pall.	Qi jiao	Aerial part	Herb	All available time	*	***	Medicine for people
TD050	Geraniaceae	*Erodium stephanianum* Willd.	/	Aerial part	Herb	All available time	*	***	Medicine for people
TD027	Geraniaceae	*Geranium wilfordii* Maxim.	/	Aerial part	Herb	All available time	*	***	Medicine for people
TD028	Guttiferae	*Hypericum bellum* Li	Zhan xin	Leaves	Shrub	All available time	**	***	Medicine for people
TD053	Guttiferae	*Hypericum forrestii *(Chittenden) N. Robson	Dan bai	Leaves	Shrub	All available time	**	***	Medicine for people
TD045	Lamiaceae	*Clinopodium megalanthum *(Diels) C. Y. Wu et Hsuan ex H. W. Li	/	Aerial part	Herb	All available time	**	***	Medicine for people
TD054	Lamiaceae	*Elsholtzia ciliata* (Thunb.) Hyland.	Da liao	Aerial part	Herb	All available time	**	***	Medicine for people
TD024	Lamiaceae	*Perilla frutescens* var. *purpurascens* (Hayata) H. W. Li	Heng	Aerial part	Herb	All available time	**	***	Food, medicine for people
TD022	Lamiaceae	*Prunella vulgaris *L.	Ye su ma zi	Aerial part	Herb	All available time	**	***	Medicine for people
TD023	Oleaceae	*Ligustrum yunguiense* B. M. Miao	Fa da xin	Leaves	Shrub	All available time	***	**	Nectar source for people
TD057	Onagraceae	*Chamerion angustifolium* (L.) Holub	/	Aerial part	Herb	All available time	**	***	Medicine for people
FD033	Plantaginaceae	*Plantago depressa* Willd.	Li zhu	Aerial part	Herb	All available time	**	***	Medicine for people
TD063	Poaceae	*Avena sativa* L.	Chi ma	Aerial part	Herb	Winter only	***	***	Crop for people
TD032	Poaceae	*Bromus catharticus *Vahl.	You ni	Aerial part	Herb	All available time	***	***	/
TD059	Poaceae	*Dactylis glomerata* L.	Ri	Aerial part	Herb	All available time	***	***	/
TD039	Poaceae	*Echinochloa crus-galli *(L.) P. Beauv.	Bei zi	Aerial part	Herb	All available time	***	***	Crop for people
TD030	Poaceae	*Hordeum vulgare* var.* coeleste* L.	/	Aerial part	Herb	All available time	***	***	Crop for people
TD061	Poaceae	*Lolium perenne* L.	Ri bu e nuo	Aerial part	Herb	All available time	***	***	/
TD031	Poaceae	*Yushania* sp.	Mai	Leaves	Tree	All available time	***	***	Material use for people
TD064	Poaceae	*Zea mays* L.	/	Aerial part	Herb	All available time	***	***	Crop for people
TD037	Polygonaceae	*Fagopyrum esculentum *Moench	/	Aerial part	Herb	Winter only	***	***	Crop for people
TD044	Polygonaceae	*Fagopyrum tataricum* (L.) Gaertn.	En ma	Aerial part	Herb	Winter only	***	***	Crop for people
TD038	Polygonaceae	*Polygonum nepalense* Meisn.	Qian niang	Aerial part	Herb	Winter only	**	***	/
TD062	Polygonaceae	*Polygonum runcinatum* Buch-Ham. ex D. Don	Ri nie xi	Aerial part	Herb	All available time	**	***	Medicine for people
TD033	Polygonaceae	*Rumex nepalensis* Spreng.	Shuo fa	Aerial part	Herb	All available time	**	***	Medicine for people
TD052	Ranunculaceae	*Cimicifuga foetida* L.	Nong nai	Leaves	Liana	All available time	*	**	Medicine for people
TD048	Ranunculaceae	*Delphinium delavayi* Franch.	Ying ku	Aerial part	Herb	All available time	***	***	/
FD019	Rosaceae	*Agrimonia pilosa* Ldb.	Man ba dai	Aerial part	Herb	All available time	**	***	Food, medicine for people
TD058	Rosaceae	*Cotoneaster franchetii *Bois	Lai du du	Leaves	Shrub	All available time	***	***	Ornament for people
TD042	Rosaceae	*Cotoneaster hissaricus* Pojark	Bi xin	Leaves	Shrub	All available time	***	***	Ornament for people
TD047	Rosaceae	*Fragaria vesca* L.	Xie su	Aerial part	Herb	All available time	**	***	Fruits for people
FD027	Rosaceae	*Geum japonicum* var. *chinense* F. Bolle	Qian ci dai	Aerial part	Herb	All available time	**	***	Medicine for people
FD059	Rosaceae	*Malus rockii Rehd.*	Suan pan zi	Fruits	Tree	All available time	***	***	Nectar source, ornament for people
TD006	Rosaceae	*Potentilla chinensis* Ser.	Zi ze	Aerial part	Herb	All available time	**	***	Medicine for people
TD001	Rosaceae	*Potentilla discolor* Bge.	Long nai	Aerial part	Herb	All available time	**	***	Medicine for people
FD046	Rosaceae	*Potentilla kleiniana *Wight et Arn.	Man ba dai	Aerial part	Herb	All available time	**	***	Medicine for people
FD024	Rosaceae	*Prinsepia utilis* Royle	Zu ni	Leaves	Shrub	All available time	***	***	Food, medicine for people
TD056	Rosaceae	*Pyrus pyrifolia* (Burm. F.) Nakai	Si da ba ji	Fruits	Tree	All available time	***	**	Fruits for people
TD003	Rosaceae	*Rosa multiflora* Thunb.	Zu	Leaves	Shrub	All available time	***	***	Fruits for people
FD040	Rosaceae	*Rubus coreanus* Miq.	You qi mi	Leaves	Shrub	All available time	***	**	Fruits for people
TD004	Rosaceae	*Rubus ellipticus* var. *obcordatus* (Franch.) Focke	/	Leaves	Herb	All available time	***	***	Fruits for people
TD002	Rosaceae	*Rubus innominatus* S. Moore	Su niang	Aerial part	Shrub	All available time	**	***	Fruits for people
TD005	Rosaceae	*Rubus niveus* Thunb.	Shi she	Leaves	Shrub	All available time	***	***	Fruits for people
TD043	Salicaceae	*Populus davidiana* Dode.	Ha xin	Leaves	Tree	All available time	**	*	Material use for people
TD035	Salicaceae	*Salix cheilophila* Schneid.	Ru	Leaves	Tree	All available time	**	*	Material use for people
TD040	Smilacaceae	*Smilax biumbellata* T. Koyama	Rie bu leng	Leaves	Liana	All available time	*	*	Medicine for people
TD055	Solanaceae	*Solanum tuberosum* L.	/	Roots	Herb	Winter only	***	***	Crop for people
TD060	Urticaceae	*Elatostema involucratum* Franch. et Sav.	Niang zhu	Leaves	Herb	All available time	**	***	Medicine for people
TD034	Urticaceae	*Pilea notata* C. H. Wright	Zhisao	Aerial part	Herb	All available time	*	***	Medicine for people
FD068	Urticaceae	*Urtica fissa* E. Pritz.	Ha qi zi	Leaves	Herb	All available time	**	***	Medicine for people
FD003	Vitaceae	*Vitis heyneana* Roem. et Schult	Yi yao	Leaves	Liana	All available time	*	***	Fruits for people

Species in this inventory are ordered by the family name alphabetically. The local name of forage plants is written using Chinese *pinyin*. * in preference and abundance represents the preference level of black-boned sheep and resource amount of the forage plants. *means low preference or low abundance level, **represents medium preference or medium abundance levels, and ***is high preference or high abundance level

Herbaceous plants account for the majority, which has a lot to do with such plants being easy to obtain and eat. Woody plants, especially trees, can only be consumed by sheep with the help of shepherds. Therefore, the local people have the habit of wearing a hatchet when grazing, to obtain the branches and leaves of the trees for the sheep to eat. Local people usually use the dry stems and leaves of these plants as the main source of feed when forage plants are lacking in the winter. This is because Fabaceae and Poaceae plants are generally recognized as suitable forage plants [61–63].

**Fig. 6:**
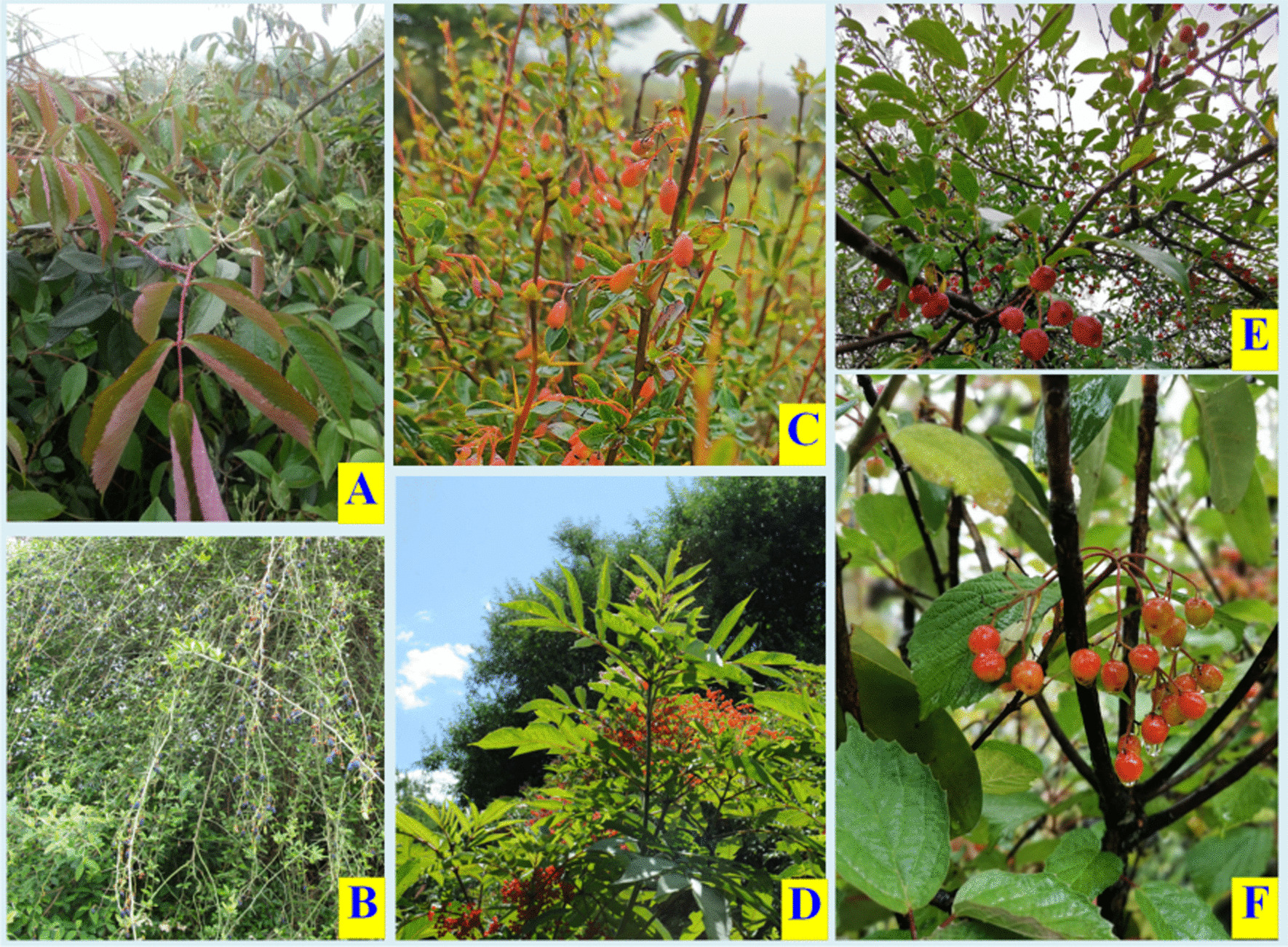
Black-boned sheep’s preferred forage plants and some wild fruits used as black-boned sheep’s forage supplements (**A** *Rosa multiflora* Thunb.; **B** *Prinsepia utilis* Royle; **C** *Berberis wilsonae* Hemsl.; **D** *Sambucus adnata* Wall. ex DC.; **E** *Malus* *rockii* Rehd.; **F** *Viburnum betulifolium* Batal.) (Photographed by Chunlin Long between July 2019 and May 2021)

Key informants are very clear about the black-boned sheep’s dietary preferences. Some plants were repeatedly mentioned during the investigation. For instance, many key informants report that black-boned sheep especially like to eat the leaves of some shrubs, such as *Rosa multiflora*, *P. utilis*, and *Berberis pruinosa* (Fig. 6). The fruits of plants such as *Sambucus adnata*, *Viburnum betulifolium*, *Elaeagnus umbellata*, and *Malus baccata* fall on the ground when they mature. Sheep are very willing to consume them, it is very beneficial for them when it comes to supplementing nutrition (Fig. 6). The key informants emphasized that the phenomenon of black-boned sheep being strong and rarely getting sick is not only related to a large amount of exercise but also may be linked to the regular consumption of these forage plants. Therefore, they always tend to gravitate to places where these plants are abundant for grazing.

The locals have a detailed understanding of the multifarious uses of various forage plants in the local, which is the experience they have accumulated in the long-term management of forests and grasslands. The key informants pointed out that many forage plants have diverse uses in the local area. For example, they use the fruits of *Rubus* plants like fruits, some people even pick the fruits of these plants to sell in the market. The locals also collect the tender leaves and shoots of plants such as *Aralia chinensis*, *T. mongolicum*, and *P. utilis* as wild vegetables. In Yunnan, many ethnic groups, including the Pumi people, traditionally use *Rhododendron decorum* flowers as food [64]. Besides, because local people have the habit of raising bees for honey, they recently discovered that the flowers of *Astragalus sinicus* and *Vicia cracca* are still effective sources of honey. Due to the plants’ high medicinal value, the locals also believe that these forage plants have a potential to develop into veterinary medicine [65, 66].

### Traditional culture related to black-boned sheep in the Pumi communities

**Fig. 7:**
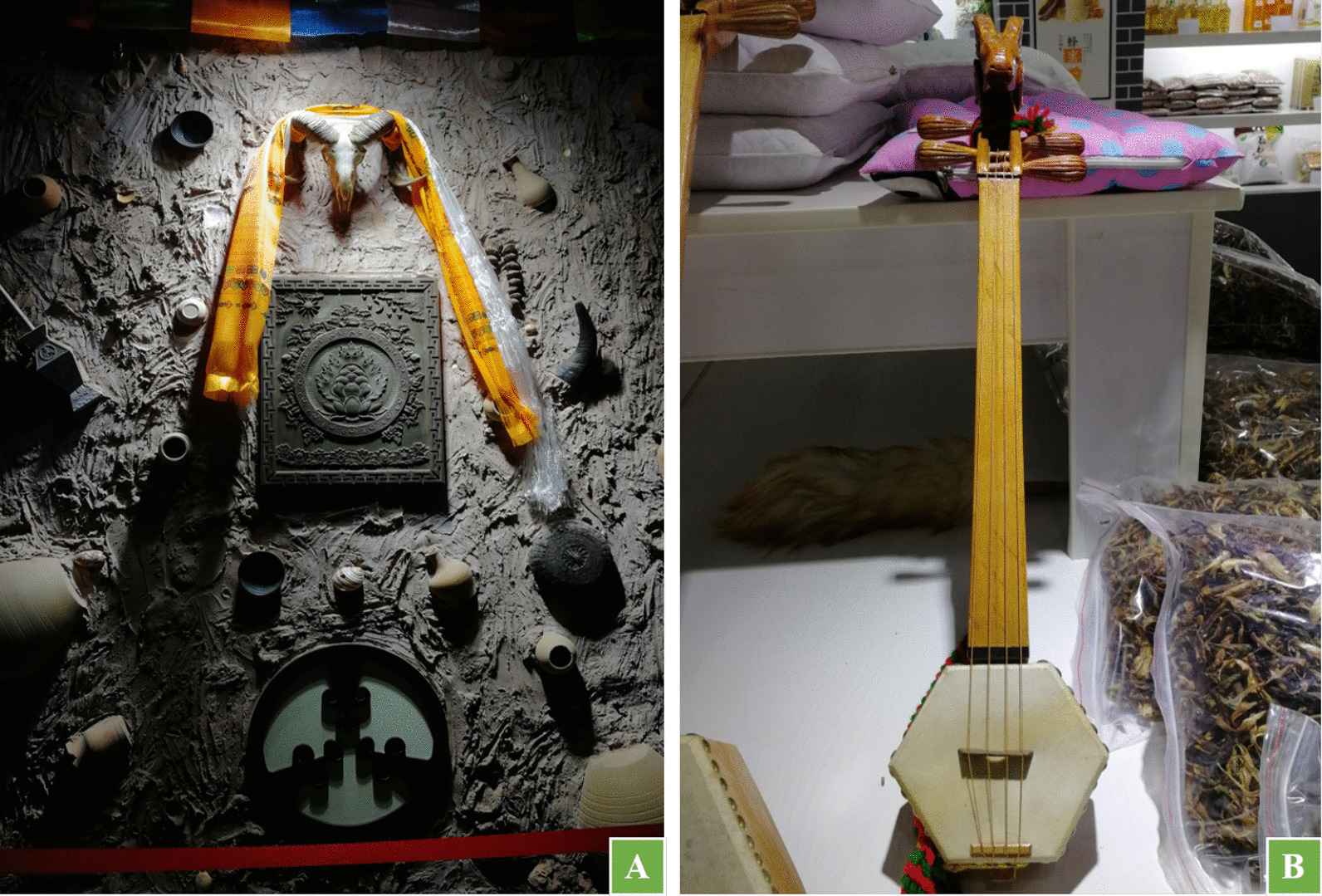
The images of sheep in the life of the Pumi (**A** Sheep totem exhibited in the Pumi Museum in Lanping County; **B** The image of sheep on the Pumi traditional musical instrument) (Photographed by Yanxiao Fan in August 2019)

Black-boned sheep is a culturally important species in Pumi communities. There are many places named after sheep in Tongdian. For example, the largest ranch there is called *Da yang chang* (大羊场) , which means a special place for sheep activities. The Pumi consider sheep as a sacred object. The image of sheep can be seen everywhere in the life of the Pumi people (Fig. 7). In local communities, the most prominent of all sheep-related cultures is *Gei Yang Zi* (给羊子), which is a significant funeral ceremony of the Pumi people. *Yang Zi* is the Pumi’s cordial name for sheep. *Gei Yang Zi* is a traditional funeral ritual passed down by the Pumi people for generations, in the rich style of ancient nomadic tribes. In this ceremony, the sheep is the absolute protagonist. The ritual generally consists of three procedures: “Sacrificing sheep,” “Guiding the way,” and “burial.”

#### Sacrificing sheep

On the second night of the death of the deceased, the family carefully selected a white and strong sheep (according to key figures, the black-boned sheep with a pure white coat is the most desirable), and the male and female are determined by the sex of the deceased. The sheep’s hooves, heads, and horns will be washed with spring water, but do not the body. Then, family members smoke the sheep with *Rhododendron* branches and leaves, remove any contamination, and thoroughly clean it. During this process, the chief priest was invited to sing “Tune for the Sheep.”

#### Guiding the way

To guide the way is to enable the dead to find their way back to their ancestors. At the beginning of the ceremony, the Pumi traditional religious figure *Shi bie* (释别) chanted the scriptures aloud. As the ceremony progressed, they fed the sheep with sacred objects such as wine and food. Under the guidance of *Shi Bie*, the sheep pointed out the migration route of his ethnic group or branch to the undead. This was so that he could return to the birthplace of his ancestors. The main message of the scripture chanted is: “Sheep are companions of the soul of the deceased, and are also their guides.” There is the companion of the sheep on the way home, and the deceased should not be afraid of any difficulties.

#### Burial

Finish reciting the “Guided Path Scripture.” At dawn, the funeral procession sets off. At the front of the team is a horse-leader with a saddle on the horse, which symbolizes the deceased’s ride. After that, one person holds a torch to guide the deceased, but also to send fire to the deceased. In the end, 8 people brought the wooden coffin to the funeral. When several people carried the bamboo basket, there were eggs, slime, and other food items, including the indispensable sheep tied with paper.

As a special cultural presentation, *Gei Yang Zi* is not only a symbol of ethnic ideology, but also a bond of ethnic spiritual reorganization. Therefore, it has profound cultural connotations. At the funeral of the Pumi people, giving the sheep is the most solemn ceremony.

### The role of *Gei Yang Zi* in the cultural identity of the Pumi

With the transformation of social life and the continuous improvement of national integration, many traditional rituals have lost their original functions. They are slowly being simplified and changed, or even disappearing. However, *Gei Yang Zi* is still a relatively complete traditional funeral custom in a minority of Pumi areas. It plays an integral part in the inheritance of spiritual culture and material culture of the ethnic group. Rather than saying that *Gei Yang Zi* is an ancient ritual, it is better to say that it is the soul of the Pumi people. This is because it is not only a medium for them to store and disseminate their traditional culture, but also a center for spiritual reorganization and memory identification. Therefore, in the future, while protecting the high-quality genetic resources of the black-bone sheep through local government propaganda, corporate conservation, and personal breeding, we must also realize the importance of protecting the traditional culture of the Pumi people on which the black-bone sheep depends.

It is obvious that indigenous cultures cannot be passed down to future generations in their original forms [67, 68]. What we can do is to keep up with the times and add relevant content to it based on the original cultural heritage. Just as the Pumi people did, they replaced ordinary white sheep with black-boned sheep with pure white fur in the ceremony of *Gei Yang Zi*. On the one hand, it is the protection of precious genetic resources, and it is also the innovative development of traditional culture. Today, *Gei Yang Zi* has become a cultural symbol of the Pumi people. From this we can see their bravery, kindness, and their yearning for a better life in the future.

## Conclusion

Our research reveals the importance of interaction between folk traditional knowledge and conservation of black-boned sheep, a precious genetic resource. On the one hand, the traditional breeding techniques, grazing methods, and a variety of forage plants used by Pumi people are of high reference value for the government and individuals to establish breeding bases and high-quality alpine pastures to protect the germplasm resources of black-boned sheep. On the contrary, the traditional funeral ceremony related to black-boned sheep formed in the process of long-term grazing is of paramount significance to the inheritance of the traditional culture of the Pumi people. The Pumi people's rich traditional knowledge of black-boned sheep is the experience accumulated in animal grazing, forest and grassland management, and economic crop cultivation. These experiences may also have crucial reference significance for the conservation of genetic resources in other ethnic communities. Therefore, this study believes that the Pumi people’s rich traditional knowledge related to black-boned sheep is not only conducive to the protection of the species but is also essential to the livelihood development and cultural heritage of the community.

## Data Availability

All data, materials, and information are collected from the study sites.
